# Supplementary material for: Modeling glioblastoma heterogeneity as a dynamic network of cell states
Source: Mol Syst Biol. 2021 Sep 16;17(9):e10105. doi: 10.15252/msb.202010105 (PMC8444284; doi:10.15252/msb.202010105)
Supplement: Supplementary file 5 — Source Data for Figure 3 [file MSB-17-e10105-s001.zip › Figure3A_sourcedata/GSEA_3065/hallmarks_state5.GseaPreranked.1623416302988/HALLMARK_BILE_ACID_METABOLISM.html]

Details for gene set HALLMARK\_BILE\_ACID\_METABOLISM[GSEA]

|  || Dataset | state5 |
| Phenotype | NoPhenotypeAvailable |
| Upregulated in class | na\_pos |
| GeneSet | HALLMARK\_BILE\_ACID\_METABOLISM |
| Enrichment Score (ES) | 0.23007333 |
| Normalized Enrichment Score (NES) | 0.82626987 |
| Nominal p-value | 0.8512111 |
| FDR q-value | 0.89928144 |
| FWER p-Value | 1.0 |
Table: GSEA Results Summary

  

Fig 1: Enrichment plot: HALLMARK\_BILE\_ACID\_METABOLISM      
 Profile of the Running ES Score & Positions of GeneSet Members on the Rank Ordered List

  

| PROBE | GENE SYMBOL | GENE\_TITLE | RANK IN GENE LIST | RANK METRIC SCORE | RUNNING ES | CORE ENRICHMENT || 1 | EFHC1 |  |  | 148 | 0.486 | 0.0420 | Yes |
| 2 | ABCA5 |  |  | 616 | 0.282 | 0.0237 | Yes |
| 3 | FADS2 |  |  | 780 | 0.245 | 0.0349 | Yes |
| 4 | IDH1 |  |  | 793 | 0.242 | 0.0628 | Yes |
| 5 | DHCR24 |  |  | 795 | 0.242 | 0.0918 | Yes |
| 6 | PEX1 |  |  | 802 | 0.241 | 0.1202 | Yes |
| 7 | HSD17B11 |  |  | 949 | 0.219 | 0.1302 | Yes |
| 8 | ATXN1 |  |  | 1062 | 0.203 | 0.1422 | Yes |
| 9 | FADS1 |  |  | 1148 | 0.191 | 0.1557 | Yes |
| 10 | LONP2 |  |  | 1460 | 0.156 | 0.1398 | Yes |
| 11 | MLYCD |  |  | 1590 | 0.142 | 0.1425 | Yes |
| 12 | SLC22A18 |  |  | 1593 | 0.142 | 0.1593 | Yes |
| 13 | NPC1 |  |  | 1642 | 0.138 | 0.1706 | Yes |
| 14 | SLC35B2 |  |  | 1715 | 0.132 | 0.1785 | Yes |
| 15 | CROT |  |  | 1731 | 0.131 | 0.1927 | Yes |
| 16 | SLC23A2 |  |  | 1778 | 0.128 | 0.2029 | Yes |
| 17 | PEX13 |  |  | 1855 | 0.122 | 0.2091 | Yes |
| 18 | CYP46A1 |  |  | 1974 | 0.113 | 0.2096 | Yes |
| 19 | ABCA2 |  |  | 1975 | 0.113 | 0.2232 | Yes |
| 20 | PEX26 |  |  | 2068 | 0.107 | 0.2259 | Yes |
| 21 | ABCA1 |  |  | 2142 | 0.103 | 0.2301 | Yes |
| 22 | GSTK1 |  |  | 2283 | 0.093 | 0.2256 | No |
| 23 | GNPAT |  |  | 2476 | 0.081 | 0.2139 | No |
| 24 | SLC29A1 |  |  | 2508 | 0.079 | 0.2199 | No |
| 25 | ABCA3 |  |  | 2662 | 0.070 | 0.2112 | No |
| 26 | AR |  |  | 2977 | 0.053 | 0.1824 | No |
| 27 | PEX16 |  |  | 3885 | 0.012 | 0.0824 | No |
| 28 | HSD17B4 |  |  | 3981 | 0.008 | 0.0727 | No |
| 29 | HACL1 |  |  | 4147 | 0.001 | 0.0543 | No |
| 30 | PEX6 |  |  | 4191 | -0.001 | 0.0496 | No |
| 31 | NEDD4 |  |  | 4399 | -0.010 | 0.0277 | No |
| 32 | CYP27A1 |  |  | 4437 | -0.012 | 0.0250 | No |
| 33 | RXRA |  |  | 4441 | -0.012 | 0.0260 | No |
| 34 | ACSL1 |  |  | 4862 | -0.028 | -0.0176 | No |
| 35 | PECR |  |  | 4978 | -0.032 | -0.0266 | No |
| 36 | ABCD3 |  |  | 5045 | -0.034 | -0.0299 | No |
| 37 | CAT |  |  | 5358 | -0.047 | -0.0591 | No |
| 38 | ISOC1 |  |  | 5369 | -0.047 | -0.0545 | No |
| 39 | SLC27A5 |  |  | 5423 | -0.049 | -0.0545 | No |
| 40 | GCLM |  |  | 5728 | -0.063 | -0.0810 | No |
| 41 | ALDH9A1 |  |  | 6006 | -0.074 | -0.1030 | No |
| 42 | FDXR |  |  | 6009 | -0.074 | -0.0943 | No |
| 43 | PNPLA8 |  |  | 6200 | -0.085 | -0.1053 | No |
| 44 | PHYH |  |  | 6255 | -0.088 | -0.1008 | No |
| 45 | PFKM |  |  | 6344 | -0.092 | -0.0995 | No |
| 46 | PEX19 |  |  | 6808 | -0.122 | -0.1367 | No |
| 47 | IDI1 |  |  | 7337 | -0.172 | -0.1751 | No |
| 48 | OPTN |  |  | 7631 | -0.208 | -0.1828 | No |
| 49 | PXMP2 |  |  | 7827 | -0.240 | -0.1757 | No |
| 50 | RBP1 |  |  | 7877 | -0.250 | -0.1510 | No |
| 51 | IDH2 |  |  | 8498 | -0.434 | -0.1681 | No |
| 52 | SCP2 |  |  | 8513 | -0.442 | -0.1164 | No |
| 53 | PRDX5 |  |  | 8836 | -0.693 | -0.0690 | No |
| 54 | SOD1 |  |  | 8848 | -0.709 | 0.0152 | No |
Table: GSEA details [plain text format]

  

Fig 2: HALLMARK\_BILE\_ACID\_METABOLISM: Random ES distribution      
 Gene set null distribution of ES for **HALLMARK\_BILE\_ACID\_METABOLISM**
